# Cultural Adaptation and Evaluation of the Perceived Nutrition Environment Measures Survey to the Mediterranean Spanish Context (NEMS-P-MED)

**DOI:** 10.3390/nu12113257

**Published:** 2020-10-24

**Authors:** Alba Martínez-García, Eva María Trescastro-López, María Eugenia Galiana-Sánchez, Cristóbal Llorens-Ivorra, Pamela Pereyra-Zamora

**Affiliations:** 1 Department of Community Nursing, Preventive Medicine and Public Health and History of Science, University of Alicante, 03690 Alicante, Spain; alba.martinez@ua.es (A.M.-G.); galiana@ua.es (M.E.G.-S.); cristobal.llorens@ua.es (C.L.-I.); pamela.pereyra@ua.es (P.P.-Z.); 2 Public Health Center of Dénia (Alicante), Valencian Community, 03700 Alicante, Spain

**Keywords:** food environment, measurement, validation study, surveys and questionnaires, perception, nutrition environment measures surveys, Spain

## Abstract

Individuals’ perceptions of their food environments are a mediator between exposure to the environment and people’s interaction with it. The Nutrition Environment Measures Surveys (NEMS) are valid and reliable measures to assess food environments. In Spain, there is no adapted instrument to measure the perceived obesogenic environment. This article aims to adapt and evaluate the Perceived Nutrition Environment Measures Survey for a Spanish context (NEMS-P-MED). The Spanish version has 32 questions to measure the perception about availability, accessibility and marketing of 3 types of environment: home, shops and restaurants. We assess feasibility, construct validity and internal consistency reliability through a sample of 95 individuals. The internal consistency was acceptable for most items (Cronbach’s alpha coefficients range from 0.6 to 0.9), similar to that of the original scale. The NEMS-P-MED has been shown to be valid and, on certain items reliable, and was useful to assess the population’s perceptions of the food environment in the home, restaurants and food stores in a Spanish context. Adapting standardized measurement tools to specific contexts to assess the perceived and observed characteristics of food environments may facilitate the development of effective policy interventions to reduce excess weight.

## 1. Introduction

In Spain, and on a global level [1,2], in recent decades there has been a considerable increase in the prevalence of overweight and obesity (OW/OB) [3]. A fundamental element for understanding the current obesity epidemic is the obesogenic environment [4,5], considered as a further determinant causing the high levels of excess weight and defined as the unhealthy environment that predisposes to excess weight gain, promoting inactivity, sedentary behaviour and/or poor diets among individuals [5,6]. Forming part of this is the food environment, described as the opportunity to obtain food, its availability, accessibility, advertising and sale [7]. Within it, food can be accessed in different ways: in the home, points of sale (food stores, supermarkets, markets), in eating establishments (bars, restaurants, canteens, take-away restaurants) and in institutions where people spend part of their day (workplaces, schools) [8].

The identification of the obesogenic environment has been conducted using quantitative methods such as observational questionnaires (audits, checklists, systematic social observation), surveys or government health reports, geographic information systems (GIS) and/or questionnaires capturing the perception of individuals [9]; qualitative methods were also used through focus groups or the photovoice technique [10,11,12,13]. The importance of determining which elements constitute these obesity-generating environments has led to the development of data collection instruments [9,14,15,16,17,18]. However, data collection instruments that consider the perception people have of their food environment are scarce [9]. Some research has shown that perception is a mediator between the exposure to the environment and the interaction of people with it [5,19]. For this reason, it is relevant to use perception-measurement approaches to complement objective measures, to understand how people perceive and interact with their food environment by considering it as a key element in addressing the problem of excess weight.

In order to measure the perceived food environment, efficient, feasible and reliable tools are necessary. Of the existing instruments, some of the most widely used to characterize the food environment have been developed in the United States for the American context by Glanz et al. [9,20], known as the Nutrition Environment Measures Survey (NEMS) [21]. There are different NEMS instruments focused on identifying different types of food environments, such as food stores [22], restaurants [23], vending machines [24] or the perceived environment [25], in order to describe them and establish an association with diet and finally with the results that this has on health. Contrary to other NEMS instruments, the “Perceived Nutrition Environment Measurement Survey” (NEMS-P) [25], created in 2015, is not an observation measure but is instead an instrument to assess the perceived food environment analyzing the interpretation and perception people have over the age of 18 regarding their food environment, focusing on food stores, restaurants and home. Additionally, it gathers information about certain food behaviours on an individual and household level of the person interviewed.

To date, this is the only instrument that assesses the perception of different types of food environment (food stores, restaurants and home) [9]. Furthermore, currently there are no questionnaires regarding the obesogenic environment adapted to the Spanish context that analyze people’s perception of their food environment. It is obvious that food habits and consumption patterns vary between countries, and therefore, the food environment of the American context is not comparable with that of Spain. Mediterranean food environments have specific characteristics, such as the diversity of food store types, with the presence of small retailers or food markets, as opposed to the high dependence on supermarket chains in the United States and other Anglo-Saxon countries [26], and also the diversity of types of restaurants. In addition, there is a different food pattern with higher consumption of fish, legumes and olive oil in southern European countries [27].

In light of the above, the aim of this study is to assess the NEMS-P instrument and culturally adapt it to the Spanish Mediterranean context.

## 2. Materials and Methods

### 2.1. Design of the Study

The NEMS-P questionnaire was culturally adapted and validated for the Spanish context, following the process described by Ramada, Serra and Delclós [28]. This process includes: (a) the cultural adaptation of the instrument, consisting in the translation, back-translation, examination by a committee of experts and the pilot testing of the scale; and (b) the validation of the instrument, through the verification of the properties that determine its reliability, validity and feasibility.

### 2.2. Development of the NEMS-P-MED

#### 2.2.1. Translation and Transcultural Adaptation

After having obtained consent from the research group that developed the NEMS-P questionnaire, the translation and transcultural adaptation was carried out. This process comprised the translation and interpretation of the original questionnaire in English into Spanish. To do this, two translations were made simultaneously by two experts. One is a native speaker and the other an English interpreter specialized in the field of health sciences.

Then, a grammatical, linguistic and semantic assessment was made of the two translations by an expert committee made up of a multi-disciplinary team: two dieticians-nutritionists (EMTL, AMG), a nurse (MEGS) and a statistician (PPZ), in order to reach an agreement on the final questions, taking into account the quality of the adaptation of the expressions and items of the questionnaire to the Spanish context.

A pilot test was carried out with the preliminary version of the questionnaire among 10 people. These participants were excluded from the main study. The pilot test of the questionnaire was conducted with the objective of assessing its correct translation, determining its feasibility, evaluating the global understanding of the questionnaire, confirming the correct choice of variables, adjusting the criteria and evaluating the formulation of the questions.

The pilot test allowed adjustments to be made to the questionnaire, including, eliminating or modifying items and thereby defining the NEMS-P-MED questionnaire. It was decided to add “MED” due to its application in the Mediterranean context, and to include foods, food stores and restaurants typical of the Mediterranean areas, such as Spain.

Furthermore, a manual for the interviewers was developed to guarantee that the collection of data through the NEMS-P-MED was carried out homogeneously by all of the interviewers.

#### 2.2.2. Modification and Final Structure of the Questionnaire

The original questionnaire has 49 fundamental items in the conceptual framework developed for constructing the instrument [25]. This framework is an extension of the “Model of Community Nutrition Environments” described by Glanz et al [21], based on updates of the evidence on the food environment [25]. The framework suggests that the interaction of the perceived and observed food environments influences the food behaviours both directly and indirectly through food purchasing behaviours (for example, frequency of purchases or planning of food shopping), the frequency of use of restaurants and the home food environment. 

In order to adapt the NEMS-P to the Spanish context, the food items consumed have been modified, adapting to the Spanish Mediterranean pattern [29,30] and to the type of food stores and restaurants characteristic of the Spanish context. As such, we included additional food items not found in the original NEMS-P: nuts, oil, legumes, meat or fish in the section of availability of food in the home. We also excluded some food items (hot dogs) from the original tool in the same section. Regarding food stores, we included food markets and small specialized stores (e.g., fruit & vegetable stores, butcheries, bakeries or fishmongers), and in restaurants, we added tapas bars.

After the pilot testing, a new item was incorporated (frequency of use of eating establishments with a wider scale) and the order of the others was modified. Furthermore, it was observed that the questionnaire was long and that the same information could be collected with less and grouped questions. For this reason, certain items were eliminated (such as: appliances do you have in your home to cook or store food) and others were grouped together. 

The final questionnaire, therefore, is composed of 32 questions that are grouped into five dimensions which are shown in Table 1. The questions have different types of responses: dichotomous (yes/no), ordinal with a Likert-type scale from 3 to 6 options depending on the dimension (degree of agreement, importance or frequency), list of categories (for example: level of education, work, civil status, level of income) and direct notation (for example: age, weight, height, address). The complete instrument is available in Table S2.

### 2.3. Data Collection and Selected Population

The data was collected in the Altabix health center in the town of Elche (Alicante, Region of Valencia). We chose this health center because it serves people from different neighborhoods of Elche with different socio-economic levels. The data were collected by three previously trained interviewers during the months of March and April 2018.

The selected population consisted of the adult population aged between 18 and 65 residing in this town. To select the sample, a population distribution similar to the population structure of the Region of Valencia was considered [31,32]. The sample was made up of 95 individuals (men 44.2% and women 55.8%), who were randomly selected from patients and persons accompanying patients who visited the Altabix health center. The selection of the individuals for the sample was based on the following criteria: the participants had to reside in family households and could not be hospitalised or residents in care homes at the time of the study. Institutionalized patients were excluded as were people who did not agree to take part, pregnant or breastfeeding women, people in the end stages of life and those with illness-related malnutrition or with a serious psychiatric disorder. The participants voluntarily consented to collaborating in the study.

### 2.4. Statistical Analysis

The psychometric properties of the questionnaire were evaluated and a descriptive analysis of the data collected was made. In order to determine the quality of the measurement of the instrument, its psychometric properties were assessed: face validity, content validity, construct validity and reliability. The face validity and the semantic equivalence of the questionnaire of its adaptation to the Spanish context were obtained through a meeting of the previously formed committee of experts after the pilot testing. The content validity was determined using the content validity index (CVI) based on the assessments carried out by the committee of experts and through a descriptive analysis of the data collected [33]. In order to determine the construct validity, the Kaiser-Meyer-Olkin (KMO) test was used, together with Bartlett’s Test of sphericity to confirm whether it is possible to conduct subsequent factor analyses with the scale. The exploratory factor analysis was conducted to identify how many factors made up each item related to the food environment which was formed by a scale.

Reliability was measured by analyzing the internal consistency, calculating the Cronbach alpha for each sub-scale. It is considered that a Cronbach alpha below 0.5 shows an unacceptable level of reliability; a value of between 0.5 and 0.6 could be considered as a poor level; if the result is between 0.6 and 0.7, there is a weak level; between 0.7 and 0.8 can be considered as an acceptable level; a level between 0.8 and 0.9 would be a good level and a value over 0.9 would be excellent [34].

In order to determine the feasibility of the scale, the time necessary to complete it was evaluated, together with the simplicity of the format and the clarity of the elements.

A descriptive analysis of the sample was carried out and the corresponding confidence intervals were calculated (CI 95%). The Chi-squared test was used to measure the relationship between perception variables of the different types of environment with socio-economic, demographic, lifestyle variables and with the incidence of overweight and obesity (OW/OB). The statistical analysis was carried out using the IBM Corp. Released 2017. IBM SPSS Statistics for Windows, Version 25.0. Armonk, NY: IBM Corp.

### 2.5. Ethical Considerations

The intervention protocol approved by the Ethical Committee of Clinical Research of the Health Department of Elche was used (UA-2017-03-22). Furthermore, all of the participants were duly informed and gave their consent for their data to be used for research purposes, in accordance with to the regulations of the Declaration of Helsinki [35,36].

## 3. Results

### 3.1. Linguistic Validation

The process of culturally adapting the questionnaire to the Spanish context involved adjustments with the recommendation of the committee of experts, changing certain terms in order to adapt them to the context in which the questionnaire was to be used. Iterative discussion between experts continued until agreement was reached that the questions were suited to the Spanish context. The number of items was modified through elimination and grouping and the response format of some of them was also changed. Some examples are question 2, where the items included were rearranged to facilitate the survey and analysis, placing fruits and vegetables in the first two items and sweets and snacks in the last one, or question 4 (type of food store) and question 11 (type of restaurant) are a grouping of two previous questions, respectively.

All these changes have enabled the face validity, semantic equivalence and the content validity for the entire instrument (CVI: 0.729) to be determined, which was found to be acceptable as the value is close to 1.

### 3.2. Feasibility

The questionnaire was found to be feasible and was accepted by both interviewers and participants. According to the comments made by the participants, it has a simple and clear format which facilitates the understanding and responses of the participants, and all items were of interest. The average time taken to respond was 20 min.

### 3.3. Construct Validity

In the validation of the original NEMS-P questionnaire [25] construct validity was based on the theoretical framework developed. In this case, an exploratory factor analysis was conducted of the construct with a multi-item scale that measured components of the food environment according to type (home, food stores, restaurants). The KMO and Barlett tests gave satisfactory results (KMO >0.5; Barlett (*p* ≤ 0.05)) in the multi-item scale that they sought to analyze, which were: 2a, 2b, 2c, 2d (KMO = 0.525; Barlett X^2^ = 41.153, *p* < 0.001), 5a, 5b, 5c, 5d, 5e, 5f, 5g (KMO = 0.547; Barlett X^2^ = 55,872, *p* < 0.001), 7a, 7b, 7c, 7d, 7e, 7f, 7g, 7h-(KMO = 0.589; Barlett X^2^ =231.107, *p* < 0.001), 9a, 9b, 9c, 9d, 9e, 9f, 9g (KMO = 0.584; Barlett X^2^ = 81.419, *p* < 0.001), 13a, 13b, 13c, 13d, 13e, 13f, 13g (KMO = 0.669; Barlett X^2^ = 132.039, *p* < 0.001).

The factor analysis was conducted independently for each question as each of them contain their own scale and measured different constructs of the food environment. The questions included in this analysis are shown in Table 2.

In Table 3 shows the results of the factor analysis. In the first question analyzed (question 2), two constructs have been found: 1) accessibility to healthy foods in the home, 2) accessibility to unhealthy foods in the home. The second question/sub-scale (question 5) differentiates three constructs of the reason for buying food. In the case of question 7 regarding the food environment in food stores, two items were differentiated: availability of health vs. unhealthy foods. In question 9, related to the marketing of foods in places of purchase, three factors were differentiated. On the other hand, in the food environment of restaurants (question 13) two factors were defined.

### 3.4. Reliability

The majority of the Cronbach alpha coefficients ranged between 0.6 and 0.9. However, there was a certain level of variability, as some results generated lower values in the items related to the accessibility to healthy options in restaurants (0.253) and the last items in motivation of selection place of purchase (0.263). The highest values were obtained in the items of accessibility to the purchase of unhealthy foods (0.9) and the availability of healthy options (0.795). Table 4 lists the Cronbach alpha values for the perceived food environment items.

### 3.5. Socio-economic and Demographic Characteristics of the Sample and the Prevalence of Excess Weight

The interviewed population had an average age of 41.54 (SD = 14.30), 44.2% were men and 55.8% were women. The majority of the participants had been educated to university level (43.2%), 64.2% were employed, 64.2% were married and in the majority of cases (47.3%) the household monthly income was between 1200 and 2700 euros. In Table S1 shows the demographic and socio-economic characteristics of the participants by sex.

In relation to the prevalence of OW/OB (Table 5), 62.1% suffered from excess weight, with this being higher in men (66.7%) than in women (58.5%). When the prevalence of excess weight is analyzed separately according to sex, men had a higher prevalence of OW/OB than women. It can be observed that there is a higher prevalence of OW/OB in people with a lower level of education (82.4%) among those with primary school studies or no education, with lower incomes (66.7%) and among married people (70.5%). There is also a statistically significant relationship (*p* < 0.05) between OW/OB and age (X^2^ = 11.113; *p* = 0.004), which increases with ageing, and between civil status (X^2^ = 9.521; *p* = 0.023), where it is higher among married people.

### 3.6. Perception of the Food Environment

#### 3.6.1. Home food Environment

The questions related to the home food environment assess the availability and accessibility to food in the home. The availability in the home of both healthy and unhealthy foods was high, as we can observe in Table 6, being very similar in people with different Body Mass Index (BMI). It should be noted that those participants with excess weight had a lower availability in the home of wholegrain cereals (bread, pasta, rice) and greater accessibility to snacks and sodas than those with a normal weight.

As for the accessibility to food in the home (Table 7), the majority of the participants often or always had access to fruit and vegetables in a bowl and/or in the refrigerator. However, this was lower in those who suffered from excess weight. On the other hand, many of the participants also always or almost always had unhealthy food in the home such as snacks and sweets in cupboards or pantries. In this case, we observed a higher accessibility to these types of foods in households where there were people with OW/OB (78% and 57.6% in OW/OB vs. 58.4% and 41.7% in normal weight people).

Regarding to the relationship between variables, there is a statistically significant association (*P* < 0.05) between the presence of different varieties of fruits in the home and the perception that the supply of fresh fruit is varied in the points of sale in the neighborhood (X^2^ = 8.066; *p* = 0.018), and between the presence of fruit in the home and its consumption (X^2^ = 15.601; *p* = 0.008). However, the case for vegetables is not the same.

In relation to unhealthy foods and excess weight, there is a statistically significant association (*P* < 0.05) between the presence of snacks in the house and the existence of OW/OB (X^2^ = 4.726; *p* = 0.030). With the rest (sweets and sodas), no association has been found.

With regard to the level of household income, there is a statistically significant relationship (*P* < 0.05) between the level of income and the presence in the home of fish (X^2^ = 9.691; *p* = 0.021) and the availability of snacks (X^2^ = 12.165; *p* = 0.007) and sweets (X^2^ = 8.624; *p* = 0.035).

#### 3.6.2. Perception of the Retail Food Environment

Insofar as food shopping, more than half of the participants (53.7%) shopped once a week and 36.8% more than once a week. Furthermore, the majority of the participants did most of their shopping in supermarkets (89.5%) and the rest in indoor or outdoor markets (10.5%). When asked about the place of purchase of fruit and vegetables, a question which allowed all of the options to be marked, the majority responded that they bought these products in supermarkets (67.4%), followed by greengrocers (46.3%) and indoor or outdoor markets (46.3%). A minority bought fruit and vegetables in small specialized stores (fruit and vegetables stores) (12.6%), 24h stores (2.1%) or local orchards (1.1%). Generally, 78.9% considered that in the places where they bought fruit and vegetables the prices were normal, without observing relevant differences between the types of store.

When shopping in a certain food store, the majority of the participants gave greater importance to the quality (77.9%), variety (73.7%) and price (58.9%) of the food than to factors related to the location of the food store close to their home (48.4%) or to places close to where they spend time or on their route (18.9%). It should be noted that, when differentiating between types of food stores, all participants who usually shopped in indoor or outdoor markets valued the quality of the food. 

On the other hand, more than 80% of the participants considered that, in their neighborhood, it was easy to find both healthy (fruit, vegetables, lean meats) and unhealthy (sweets, snacks and sodas or sugary drinks) foods. However, when divided into the type of food store frequented (Table 8), the participants perceived that more marketing techniques were used for promoting unhealthy foods in supermarkets (58.8%) than in markets (10%). Furthermore, they also confirmed that they perceived that there was more promotion of unhealthy foods (58.8%) than that of healthy foods (22.4%). Moreover, the majority of them noticed marketing strategies used in supermarkets to facilitate the purchase of unhealthy foods (unhealthy foods are usually placed at the beginning or the end of the aisles (63.5% agree); unhealthy foods are close to the checkouts (76.5%).

With regard to the associations between variables, we can observe a statistically significant relationship (*P* < 0.05) between the type of usual store and age (X^2^ = 8.429; *p* = 0.015), whereby the purchasing in indoor and/outdoor markets increases as age increases, particularly prevalent among adults over 50 years old. A statistically significant association (*P* < 0.05) can also be observed between the usual type of store and the purchase of tinned/frozen fruit and vegetables (X^2^ = 10.428; *p* = 0.005) and sodas (X^2^ = 10.800; *p* = 0.005), whereby it is perceived to be easier to find tinned/frozen fruit and vegetables and sodas in supermarkets or hypermarkets than in markets. 

Furthermore, there is a relationship (X^2^ = 13.890; *p* = 0.008) between the use of marketing techniques depending on the food store (location and promotion of food products), being more frequent in supermarkets than in markets. At the same time, there is a statistically significant relationship (X^2^ = 12.090; *p* = 0.017) between those who perceive the presence of food marketing and the fact that they do not buy those products located at the checkouts, for example.

#### 3.6.3. Perception of the Food Environment in Restaurants

Of the total participants (*n* = 95) there were seven people who did not usually visit bars and restaurants. Therefore, only those who did visit them responded to the corresponding questions (*n* = 88). Most of the participants usually visited à la carte or set menu restaurants (41.1%), followed by fast food restaurants (29.5%) and finally other types of establishments, such as cafeterias (22.1%). It can also be observed that there was a higher percentage of participants who visited restaurants/bars less frequently (never or almost never), whether they were fast food restaurants (55.8%), à la carte/set meal restaurants (52.6%), or others such as cafeterias, bars or tapas bars (45.3%). We can also observe a gradient in the prevalence of obesity (Table 9), which was found to be higher in those participants who visit different collective catering establishments less often (67.9% fast food; 68.0% à la carte/set meal; 65.1% others such as cafeterias or tapas bars).

In Table 10, we can observe the perception of the food environment in eating establishments by the type of restaurant visited. A large part of the participants perceive that the healthy options are more expensive in all types of restaurant, that the presence of healthy options is greater in à la carte/set meal restaurants and that the greatest promotion of unhealthy options occurs in fast food restaurants. Furthermore, it should be pointed out that a large part of the participants in the OB/OW category did not perceive that the promotion of unhealthy options used signs or posters in à la carte/set meal restaurants (33.9%) and other types such as cafeterias, bars or tapas bars (33.3%).

A statistically significant association (*P* < 0.05) can be observed between the type of restaurant and the availability of healthy options on the menu (highlighting that this occurs to a greater extent in à la carte or set meal restaurants) (X^2^ = 20.141; *p* = 0.010), the promotion and marketing of unhealthy foods in the restaurant (more frequently in fast food restaurants) (X^2^ = 25.507; *p* = 0.001), age (the largest consumers of fast food restaurants are the youngest participants) (X^2^ = 15.838; *p* = 0.003) and the level of income (people with higher incomes visit à la carte/set menu restaurants more frequently) (X^2^ = 23.591; *p* = 0.001). With respect to income, we can highlight that the participants with lower incomes give greater value to the price of a restaurant with respect to the other variables for selecting the establishment (X^2^ = 20.968; *p* = 002).

## 4. Discussion

This study adapted and evaluated the Perceived Nutrition Environment Measures Survey for the Spanish population. The Spanish version of the NEMS-P questionnaire is feasible for the Spanish context. It has a lower number of questions than the original version and has required the modification of some of them in order to improve its adaptation. Furthermore, the questionnaire gathers the perception of three types of environment (the home, food stores and restaurants). This implies that, depending on the interest of the researcher, it is possible to complete only one section, as the questions are divided into sections that refer to each of these environments independently.

The version of the NEMS-P-MED for the Spanish context has proved to be valid and, in certain items, reliable. The internal consistency was acceptable for the majority of the items, similar to that of the original scale [25]. However, the organisation of the factors in certain items has been conducted differently to the original, following the groupings established in the exploratory factor analysis. In this case, the availability of healthy foods was divided into two groups (ease of purchase of fruit/vegetables vs. low-fat foods). In addition, the original study did not analyze the group of items relating to the availability of unhealthy foods (sweets, soft drinks and snacks), and in this case it did, obtaining a high degree of reliability. Also, the grouping of items related to the location and promotion of unhealthy food was separated into the perception of the location and purchase of food located in these places. In the case of the restaurant food environment, they were also reorganized differently, according to availability, accessibility and promotion of food. The lowest values are those obtained in the accessibility to healthy options in restaurants and the motivation to buy in a food store. Although factor analysis includes these groupings, they may be different from each other and do not measure the same dimension. Low values are likely to indicate some questions are not strong measures of that dimension or that they measure aspects of that dimension that overlap with other dimensions. Further refinement of questions may improve these values. Even though some of these values are not very high, the data are collected that are intended to represent the conceptual framework of the questionnaire, and that make up the obesogenic food environment.

With regard to the information obtained in the section on the home food environment, we can observe that the availability and accessibility to healthy and unhealthy foods was high in the majority of homes, with it being higher in terms of unhealthy foods (savoury snacks, sweets and sodas) in the homes of people in the OB/OW category and lower income levels. These results coincide with the data obtained from the original questionnaire and with previous researchers who found significant associations between the availability and variety of foods perceived with the consumption of fruit and vegetables [25,37,38,39]. Similarly, associations are established with the type of foods in the home and the level of income, with a lower presence of fish and a higher presence of snacks and sweets in the lowest income households. This can be explained by the price of these foods, as fish is less accessible as it is more expensive than other foods, and ultra-processed foods are cheaper [4]. Similar results are also obtained to those of the Spanish National Health Survey of 2017, where the groups with the lowest socio-economic level consume less fruit and vegetables and a higher amount of sodas [3]. In Spain as a whole, over the last few decades there has been a shift from eating and having in the home fresh, local and seasonal foods to having access to a greater supply of cheap foods form different origins throughout the year and energy-dense foods poor in nutrients [40].

On the other hand, in the section referring to the food environment in food stores, it is worth pointing out that supermarkets are most visited for the purchase of foods than small traditional specialized grocery stores (for example, greengrocers or butchers) and indoor and outdoor markets, coinciding with the results obtained in other studies carried out in Spain [41,42]. A statistically significant relationship has been established between the type of store usually visited, age and the level of income, being more frequent purchases in indoor and/outdoor markets among older people with higher incomes. This can be explained because the majority of people who continue shopping in markets do so because they have been doing it all of their lives as years ago, the markets and specialized stores were the most abundant type of stores in the Spanish context [26]. In the choice of food store, the majority of the participants gave more importance to quality, variety and price than to the proximity of the food store to their home or if it was on their way to other places where they spend time. This may be because, contrary to British or American cities, Spanish/Mediterranean cities and neighborhoods have a compact urban nucleus where it is easy to walk or access food stores without transport, as highlighted in previous studies [26,43]. On the other hand, the majority of the population studied perceived that there was wide availability of and accessibility to fruit and vegetables, considering that the supply was varied and of a high quality without obtaining a great difference in price between the different types of stores. Furthermore, the data obtained in this study on the availability of healthy foods are similar to a previous study which analyzes the distinctive factors of the Mediterranean context in the food shopping environment in Spain [26]. Unlike the British and American environments, the closeness and availability of healthy foods in food stores is greater in the Spanish context [43]. With respect to the accessibility to food in stores, a large part of the participants perceived that it was easy to find both healthy and unhealthy foods. However, they confirmed that they perceived more promotion of unhealthy foods than healthy foods, and that this was principally in supermarkets. Although the evidence is not clear, previous studies find similar results which affirm that supermarkets contribute to the increase in the consumption of unhealthy processed foods, as they have a greater supply and promotion than local, specialized stores or markets [44,45,46].

With respect to the section of the questionnaire referring to the food environment in restaurants, the participants perceive that the healthy options are more expensive in any type of restaurant, therefore they are less accessible. Furthermore, they observe that there is a higher presence of healthy foods in à la carte/set meal restaurants and that the promotion of unhealthy options is greater in fast food restaurants. Similarly, in previous studies, restaurants are perceived as one of the principal barriers to obtaining a healthy weight, due to the scarce supply of healthy foods and the marketing of unhealthy products [23,47]. This study finds that the largest consumers of fast food restaurants are the youngest participants, coinciding with the results of previous research [48]. These types of restaurants have increased in number and represent a more economic but less healthy supply [49].

It is worth pointing out that, in the three types of environment, the level of income is related to OW/OB of the population and to the choice or presence of unhealthy foods in the home, food stores or restaurants. Globally, we can observe that there is an association between the socio-economic level and diet and excess weight [50,51,52,53,54,55]. Previous studies carried out among the North American population show that populations with lower incomes have a greater probability of being exposed to unhealthy foods [56,57]. This also occurs in Spain, according to previous studies which conclude that socially disadvantaged people have a higher exposure to unhealthy foods due to their place of residence [41,58,59,60]. Moreover, the places of sale of food characterised by their wide accessibility to ultra-processed, affordable foods which are continuously promoted in the media, lead to unhealthy food behaviours in those with least material and psychosocial resources [61]. Therefore, we can affirm that economic inequalities influence the food environment of the population. However, these inequalities in health could be avoidable and/or reversible through efficient public policies. In order to address the high prevalence of OW/OB through public policies, it is necessary to contemplate the environment using an inter-disciplinary approach by comparing observational and perception methods in order to gain a greater understanding and uniformity of the characterization of the food environment and its influence on excess weight [15,20,62].

Finally, this study also has a series of limitations. First, some variables have a low internal consistency. Consequently, there remains an opportunity to further refine questions to improve internal consistency, whilst still adhering to the conceptual framework of the original questionnaire. However, the results obtained in the original validation are similar [25]. On the other hand, the test-retest reliability could not be carried out as the majority of the participants did not send the questionnaire after two weeks as requested. There is therefore a need for a test-retest reliability. Moreover, as the survey was carried out in a health center, it is assumed that there will be a higher percentage of the sample with an illness. Nevertheless, to reduce this bias, companions were also interviewed. Furthermore, in future research, in order to gain a better understanding of the food environment, the study sample should be extended and comparisons between differentiated neighborhoods should be made in accordance with the socio-economic level, which are then compared with the data collected through the complementary NEMS-S and NEMS-R instruments. The NEMS-S-MED questionnaire has recently been adapted to the Spanish context [63], so a comparison with it would be possible. Regarding the NEM-R-MED questionnaire, it is in its first stages of cultural adaptation to the Spanish Mediterranean context, and in the future it would also be available to assess the restaurant environment and compare it with the results obtained on people’s perceptions through the NEMS-P-MED.

## 5. Conclusions

The adapted NEMS-P-MED instrument is feasible and useful for evaluating the perceived food environments in the Spanish context. Future research could apply this instrument in other Mediterranean contexts, and compare its results with objective measures such as the recent adapted NEMS-S-MED survey. The adaptation of standardized measurement instruments to evaluate context-specific features and comparing perceptive with objective measures would facilitate understanding of key contextual determinants of food environments. Gaining a better understanding of the food environment and its effects at community and individual levels would promote and strengthen the development and assessment of effective public health interventions to improve food environment and reduce excess weight.

## Figures and Tables

**Table 1:** Development of the Perceived Nutrition Environment Measures Survey for a Spanish context (NEMS-P-MED) questionnaire.

Dimensions	Number of Questions	Variables Included
Home food environment	2	Availability and accessibility to food in the home
Food shopping	7	Frequency of shopping, type of shop where the majority of the purchases are made (supermarket, local shop, indoor market/outdoor market, cooperative), availability and accessibility to healthy and unhealthy foods, perception of prices and marketing of foods, reason for shopping (quality, variety, price, proximity) and the transport used to visit the establishment.
Eating establishments	4	Type of restaurant (set meals/à la carte menu, fast food and others), availability, accessibility and promotion of healthy vs unhealthy foods.
Opinions and habits in terms of food	4	Concern about the nutritional content of food, frequency of consumption of food, important factors when purchasing food or going to a restaurant/bar.
General questions about the home and the person	15	Sex, age, height, weight, place of birth, civil status, employment situation, level of education, general state of health, smoking habits, physical exercise, net income of the household, total number of people living in the household, type of neighborhood.

**Table 2:** Constructs and multi-items scale questions included in the exploratory factor analysis.

Questionnaire Dimensions	Multi-Item Scale Questions
(A) Home food environment	
(Question 2) Accessibility to food in the home	4
(B) Food environment in the place of the food purchase	
(Question 5) Reason for selecting the usual point of sale:	7
(Question 7) Accessibility to food in the place of purchase;	8
(Question 9): Location and promotion of food	7
(C) Food environment in restaurants	
(Question 13) Availability, accessibility and promotion of healthy foods in restaurants	7

**Table 3:** Exploratory factor analysis by factors according to the type of food environment contained in the NEMS-P-MED questionnaire.

	HOME					FOOD STORE						RESTAURANTS		
Item	Availability		Store motivation			Availability			Marketing			Availability, Accessibility and Marketing		
	F1	F2	F1	F2	F3	F1	F2	F3	F1	F2	F3	F1	F2	F3
2A	0.719													
2B	0.848													
2C		0.894												
2D		0.809												
5A			0.826											
5B			0.712											
5C			0.635											
5D				0.849										
5E				0.806										
5F					0.867									
5G					0.528									
7A						0.890								
7B						0.860								
7C						0.678								
7D							0.755							
7E							0.871							
7F								0.884						
7G								0.874						
7H								0.905						
9A									0.836					
9B									0.356					
9E									0.868					
9F										0.852				
9D										0.830				
9C											0.751			
9G											0.737			
13A												0.729		
13C												0.745		
13B													0.789	
13E													0.643	
13G													0.375	
13F														0.809
13D														0.759

Extraction method: analysis of main components. Rotation method: Varimax with Kaiser standardization. F = Factor.

**Table 4:** Cronbach alpha values and ranges for composite items that evaluate perceived food environments.

Construct	Multi-Item Scale Question	Range of Items in Question	Number of Items Analyzed	Scale Range	α
**Home Food Environment**					
Accessibility to healthy food	At home, how often you have... -fruits and vegetables in the fridge (2A) -fruit available in a bowl (2B)	1–4	2	1–4	0.425
Accessibility to unhealthy food	In your house, how often you have... -ice creams, cakes, pastas or sweets (2C) -snacks in closets or pantry (2D)	1–4	2	1–4	0.654
**Perceived Retail Food Environment**					
Motivation selection place of purchase	-Proximity to home (5A) -Proximity or on the way to passing sites (5B) -Friends or family buy there (5C)	1–7	3	1–4	0.445
	-Variety of foods (5D) -Food quality (5E)	1–7	2	1–4	0.602
	-Food prices (5F)-Access in public transport (5G)	1–7	2	1–4	0.263
Accessibility buying healthy foods	It is easy to buy/find: -Fresh fruits and vegetables (7A) -Varied offer (7B) -Canned fruit and vegetables (7C)	1–8	3	1–5	0.756
	Easy to buy low-fat products (7D) and lean meats (7E)	1–8	2	1–5	0.592
Accessibility buying unhealthy products	It is easy to buy -Sweets (7F) -Snacks (7G) -Soft drinks or other sugary drinks (7H)	1–8	3	1–5	0.900
Placementof unhealthy foods	Placement of unhealthy foods -end or start of aisles (9C) -line of boxes (9G)	1–7	2	1–5	0.376
	Buying food placed in -line of boxes (9D) -shelves at eye level (9F)	1–7	2	1–5	0.654
Promotion of food	-Promoting Healthy options (9A) -Nutrition information (9B) -Promoting Unhealthy options (9E)	1–7	3	1–5	0.603
**Perceived Food Environment in Restaurants**					
Availability of healthy options	There are many healthy menu options in the restaurant (13A) It is easy to find healthy fruit and vegetable options in the restaurant (13C)	1–7	2	1–5	0.795
Accessibility to healthy options	Difficult to find a healthy option (13B) Promoting Unhealthy Options (13E) Healthy choices are more expensive (13G)	1–7	2	1–5	0.253
Promoting healthy options	Promoting Healthy Options (13F) Nutritional Information (13D)	1–7	2	1–5	0.514

**Table 5:** Prevalence of obesity and overweight related to sociodemographic, by sex.

	OW/OB ^1^			Men			Women		
	%	N	CI ^2^ 95%	%	N	CI ^2^ 95%	%	N	CI ^2^ 95%
**Sex**	62.1	59	[52.3–71.9]	66.7	28	[52.4–80.9]	58.5	31	[45.2–71.8]
**Age**									
Young adults (18–39 years)	45.5	20	[30.7–60.2]	55.6	10	[32.6–78.5]	38.5	10	[19.8–57.2]
Adults (40–49 years)	66.7	14	[46.5–86.8]	70.0	7	[41.6–98.4]	63.6	7	[35.2–92.1]
Older adults (>50 years)	83.3	25	[70.0–96.7]	78.6	11	[57.1–100.1]	87.5	14	[71.3–103.7]
**Education level**									
Uneducated/ School	82.4	14	[64.2–100.5]	80.0	4	[44.9–115.1]	83.3	10	[62.2–104.4]
High School	58.8	10	[35.4–82.2]	50.0	3	[10.0–90.0]	63.6	7	[35.2–92.1]
Vocational education	75	15	[56.0–94.0]	75.0	9	[50.5–99.5]	75.0	6	[45.0–105.0]
University	48.8	20	[33.5–64.1]	63.2	12	[41.5–84.8]	36.4	8	[16.3–56.5]
**Employment**									
Full-time employment	63.8	30	[50.1–77.6]	70.8	17	[52.6–89.0]	56.5	13	[36.3–76.8]
Part-time employment	50.0	7	[23.8–76.2]	50.0	1	[−19.3–119.3]	50.0	6	[21.7–78.3]
Unemployed looking for a job	66.7	4	[28.9–104.4]	100	2	[100,100]	50.0	2	[1.0–99.0]
Unemployed not looking for a job (retired...)	64.3	18	[46.5–82.0]	57.1	8	[31.2–83.1]	71.4	10	[47.8–95.1]
**Marital status**									
Married	70.5	43	[59.0–81.9]	76.7	23	[61.5–91.8]	64.5	20	[47.7–81.4]
Separated, divorced	100	2	[100,100]	0	0	[0,0]	100	2	[100,100]
Widow/widower	75	3	[32.6–117.4]	0	0	[0,0]	100	3	[100,100]
Single	39.3	11	[21.2–57.4]	45.5	5	[16.0-74.9]	35.3	6	[12.6–58.0]
**Income**									
<1200 €/month	66.7	16	[47.8–85.5]	57.1	4	[20.5–93.8]	70.6	12	[48.9–92.2]
1200–2700	66.7	30	[52.9–80.4]	75	15	[56.0–94.0]	60.0	15	[40.8–79.2]
>2700	57.9	11	[35.7–80.1]	72.7	8	[46.4–99.0]	37.5	3	[4.0–71.0]

^1^ OW/OB = Overweight/Obesity; ^2^ CI = Confidence Interval.

**Table 6:** Food availability at home over the past week, according to BMI.

	Total (*n* = 95)			Normal Weight (*n* = 36)			OW/OB (*n* = 59)		
	%	N	CI ^1^ 95%	%	N	CI ^1^ 95%	%	N	CI ^1^ 95%
**Healthy Foods**									
Fruits	97.9	93	[95.0–100.8]	94.4	34	[87.0–101.9]	100	59	[100.0–100.0]
Vegetables	98.9	94	[96.9–101.0]	97.2	35	[91.9–102.6]	100	59	[100.0–100.0]
Whole milk	18.9	18	[11.1–26.8]	25.0	9	[10.9–39.1]	15.3	9	[6.1–24.4]
Semi-skimmed/skimmed milk	80.0	76	[72.0–88.0]	77.8	28	[64.2–91.4]	81.4	48	[71.4–91.3]
Refined bread	78.9	75	[70.7–87.1]	69.4	25	[54.4–84.5]	84.7	50	[75.6–93.9]
Whole bread	50.5	48	[40.5–60.6]	58.3	21	[42.2–74.4]	45.8	27	[33.1–58.5]
Refined rice and pasta	97.9	93	[95.0–100.8]	97.2	35	[91.9–102.6]	98.3	58	[95.0–101.6]
Whole-grain rice and pasta	17.9	17	[10.2–25.6]	27.8	10	[13.1–42.4]	11.9	7	[3.6–20.1]
Lean meat	94.7	90	[90.2–99.2]	91.7	33	[82.6-100.7]	96.6	57	[92.0–101.2]
Fresh or frozen fish	72.6	69	[63.7–81.6]	83.3	30	[71.2–95.5]	89.8	53	[82.1–97.6]
Legumes	86.3	82	[79.4–93.2]	88.9	32	[78.6–99.2]	84.7	50	[75.6–93.9]
**Unhealthy Foods**									
Appetizers	73.7	70	[64.8–82.5]	61.1	22	[45.2–77.0]	81.4	48	[71.4–91.3]
Sweets, biscuits and/or pastries	88.4	84	[82.0–94.9]	88.9	32	[78.6–99.2]	88.1	52	[79.9–96.4]
Soft drinks	50.5	48	[40.5–60.6]	41.7	15	[25.6–57.8]	55.9	33	[43.3–68.8]
Diet soft drinks	53.7	51	[43.7–63.7]	47.2	17	[30.9–63.5]	57.6	34	[45.0–70.2]

^1^ CI = Confidence Interval.

**Table 7:** Accessibility to food at home, according to BMI.

	NORMAL WEIGHT (n = 36)			OW/OB (*n* = 59)		
	Never/Hardly Ever % (*n*) [CI^1^ 95%]	Sometimes % (*n*) [CI^1^ 95%]	Often/Always % (*n*) [CI^1^ 95%]	Never/Hardly Ever % (*n*) [CI^1^ 95%]	Sometimes % (*n*) [CI^1^ 95%]	Often/Always % (*n*) [CI^1^ 95%]
Accessibility to healthy foods						
Fruits in a bowl or countertop	11.1 (4) [0.8–21.4]	13.9 (5) [2.6–25.2]	75 (27) [60.9–89,1]	11.9 (7) [3.6–20.1]	25.4 (15) [14.3–36.5]	62.7 (37) [50.4–75.1]
Fruits and vegetables in the fridge	2.8 (1) [−2.6–8.1]	19.4 (7) [6.5–32.4]	77.8 (28) [64.2–91.4]	13.6 (8) [4.8–22.3]	27.1 (16) [15.8–38.5]	59.3 (35) [46.8–71.9]
Accessibility to unhealthy foods						
Snacks or appetizers in closet or pantry	19.4 (7) [6.5–32.4]	22.2 (8) [8.6–35.8]	58.4 (21) [42.2–74.4]	10.2 (6) [2.5–17.9]	11.9 (7) [3.6–20.1]	78.0 (46) [67.4–88.5]
Sweets, pastries, ice cream, cakes...	36.1 (13) [20.4–51.8]	22.2 (8) [8.6–35.8]	41.7 (15) [25.6–57.8]	20.3 (12) [10.1–30.6]	22 (13) [11.5–32.6]	57.6 (34) [45.0–70.2]

^1^ CI = Confidence Interval.

**Table 8:** Perception of food availability and marketing use in the food environment in food stores, by type of frequented store.

	Scale Range	Supermarket/Hypermarket			Market		
		%	N	CI ^1^ 95%	%	N	CI ^1^ 95%
*Food Availability*							
Availability of healthy options: -Fresh fruits and vegetables -Canned/frozen fruits and vegetables -Low-fat products (such as lean meats, dairy)	1–3	91.8 81.2 83.5	78 69 71	[85.9–97.6] [72.9–89.5] [75.6–91.4]	100 50.0 80.0	10 5 8	[100.0–100.0] [19.0–81.0] [55.2–104.8]
Availability of unhealthy options -Sweets and pastries -Snacks -Soft drinks	1–3	95.3 95.3 92.9	81 81 79	[90.8–99.8] [90.8–99.8] [87.5–98.4]	90.0 90.0 70.0	9 9 7	[71.4–108.6] [71.4–108.6] [41.6–98.4]
*Food Marketing*							
Presence of signs that encourage me to buy healthy foods	1–3	22.4	19	[13.5–31.2]	0	0	[0.00–0.00]
Most packaged foods include nutritional information	1–3	82.4	70	[74.2–90.5]	50.0	5	[19.0–81.0]
Presence of signs encouraging you to buy unhealthy foods	1–3	58.8	50	[48.4–69.3]	10.0	1	[−8.6–28.6]
Foods near the box line are often unhealthy foods	1–3	76.5	65	[67.5–85.5]	NA ^2^		
Unhealthy foods are usually at the beginning or end of the aisles	1–3	63.5	54	[53.3–73.8]	NA ^2^		
*Food shopping habits*							
I usually buy food that is near the box line	1–3	15.3	13	[7.6–22.9]	NA ^2^		
I usually buy things that are placed at eye level on the shelves	1–3	31.8	27	[21.9–41.7]	NA ^2^		

^1^ CI = Confidence Interval.; ^2^ NA = Not applicable.

**Table 9:** Prevalence of OW/OB according to the most frequented type of restaurant.

	Fast Food			Menu Restaurant			Others (café, *tapas*)		
	%	N	CI ^1^ 95%	%	N	CI ^1^ 95%	%	N	CI ^1^ 95%
Never/hardly ever	67.9	36	[55.4–80.5]	68.0	34	[55.1–80.9]	65.1	28	[50.9–79.4]
Each 15 days	75.0	3	[32.6–117.4]	77.8	7	[50.6–104.9]	80.0	4	[44.9–115.1]
1 time/week	53.3	16	[35.5–71.2]	45.2	14	[27.6–62.7]	64.7	22	[48.6–80.8]
More than 3 times a week	50.0	4	[15.4–84.6]	80.0	4	[44.9–115.1]	38.5	5	[12.0–64.9]

^1^ CI = Confidence Interval.

**Table 10:** Perception of the food environment of restaurants, by type of restaurant frequented.

	Scale Range	Fast Food (*n* = 28)			Menu Restaurant (*n* = 39)			Others (café, tapas) (*n* = 21)		
		%	N	IC ^1^ 95%	%	N	IC ^1^ 95%	%	N	IC ^1^ 95%
Availability of healthy options -There are many healthy options on the menu -It is easy to find options that contain fruit and vegetables	1–3	25 25	7 7	[9.0–41.0] [9.0–41.0]	53.8 48.7	21 19	[38.2–69.5] [33.0–64.4]	42.9 38.1	9 8	[21.7–64.0] [17.3–58.9]
Accessibility to healthy choices - Healthy choices are more expensive	1–3	71.4	20	[54.7–88.2]	64.1	25	[49.0–79.2]	61.9	13	[41.1–82.7]
Promoting healthy options	1–3	10.7	3	[−0.7–22.2]	5.1	2	[−1.8–12.1]	4.8	1	[−4.3–13.9]
Promoting unhealthy options	1–3	64.3	18	[46.5–82.0]	17.9	7	[5.9–30.0]	28.6	6	[9.2–47.9]
Nutritional information on the menu	1–3	3.6	1	[−3.3–10.4]	7.7	3	[−0.7–16.1]	9.5	2	[−3.0–22.1]

**^1^ CI = Confidence Interval.**

## Supplementary Materials

The following are available online at https://www.mdpi.com/2072-6643/12/11/3257/s1, Table S1: Socioeconomic and demographic characteristics of the participants, by sex. Table S2: NEMS-P-MED Questionnaire.
