# Supplementary material for: Cultural Adaptation and Evaluation of the Perceived Nutrition Environment Measures Survey to the Mediterranean Spanish Context (NEMS-P-MED)
Source: Nutrients. 2020 Oct 24;12(11):3257. doi: 10.3390/nu12113257 (PMC7693738; doi:10.3390/nu12113257)
Supplement: Supplementary file 1 [file nutrients-12-03257-s001.pdf]

Table S1. Socioeconomic and demographic characteristics of the participants, by sex.

	Total (n=95)			Men (n=42)			Women (n=53)		
	%	N	CI 95%	%	N	CI 95%	%	N	CI 95%
**Education level**									
Uneducated/ School	17.9	17	[10.2-25.6]	11.9	5	[2.1-21.7]	22.6	12	[11.4-33.9]
High School	17.9	17	[10.2-25.6]	14.3	6	[3.7-24.9]	20.8	11	[9.8-31.7]
Vocational education	21.1	20	[12.9-29.3]	28.6	12	[14.9-42.2]	15.1	8	[5.5-24.7]
University	43.2	41	[33.2-53.1]	45.2	19	[30.2-60.3]	41.5	22	[28.2-54.8]
**Employment**									
Full-time employment	49.5	47	[39.4-59.5]	57.1	24	[42.2-72.1]	43.4	23	[30.1-56.7]
Part-time employment	14.7	14	[7.6-21.9]	4.9	2	[-1.7-11.2]	22.6	12	[11.4-33.9]
Unemployed looking for a job	6.3	6	[1.4-11.2]	4.8	2	[-1.7-11.2]	7.5	4	[0.4-14.7]
Unemployed not looking for a job (retired,...)	29.5	28	[20.3-38.6]	33.3	14	[19.1-47.6]	26.4	14	[14.5-38.3]
**Marital status**									
Married	64.2	61	[54.6-73.9]	71.4	30	[57.8-85.1]	58.5	31	[45.2-71.8]
Separated, divorced	2.1	2	[-0.8-5.0]	0	0	[0.00-0.00]	3.8	2	[-1.4-8.9]
Widow/widower	4.2	4	[0.2-8.2]	2.4	1	[-2.2-7.0]	5.7	3	[-0.6-11.9]
Single	29.5	28	[20.3-38.6]	26.2	11	[12.9-39.5]	32.1	17	[19.5-44.6]
**Income**									
<1200 €/month	25.3	24	[16.5-34.0]	16.7	7	[5.4-27.9]	32.1	17	[19.5-44.6]
1200-2700	47.3	45	[37.3-57.4]	47.6	20	[32.5-62.7]	47.2	25	[33.7-60.6]
>2700	27.4	26	[18.4-36.3]	26.2	11	[12.9-39.5]	15.1	8	[5.5-24.7]

Table S2. NEMS-P-MED Questionnaire

Since the adaptation has been for the Spanish Mediterranean context, the questionnaire is presented in the Spanish language to which it has been translated, adapted and evaluated.

CUESTIONARIO NEMS-P-MED

Número de identificación: Fecha: / /

NEMS-P-Med. Cuestionario sobre percepción del entorno alimentario
Instrucciones para completar el cuestionario
Nos gustaría conocer su opinión sobre la alimentación en la zona donde usted reside. Por favor, responda a las siguientes preguntas acerca de su vecindario, su hogar y sobre usted.<br><br>Este cuestionario es para personas de 18 años o más, que además sean responsables de buena parte o de la totalidad de las compras de alimentos para su hogar.

A. Entorno alimentario en casa

**1. Indique si, de la siguiente lista de alimentos, los tuvo o no en casa la semana pasada…**	**Sí**	**No**
a. Frutas		
b. Verduras		
c. Dulces, bollería o galletas		
d. Aperitivos de patatas fritas o snacks		
e. Embutido		
f. Refrescos normales		
g. Refrescos light		
h. Pasta, arroz, harinas refinadas		
i. Pasta, arroz, harinas integrales		
j. Pan blanco		
k. Pan integral		
l. Legumbres		
m. Carne (pollo, cerdo, ternera, conejo…)		
n. Pescado fresco o congelado		
o. Leche entera		
p. Leche semi o desnatada		

**2. Indique con qué frecuencia tiene en casa…**	**Nunca o casi nunca**	**A veces**	**A menudo / Casi siempre**	**Siempre**
a. Frutas y verduras en el frigorífico				
b. Frutas y Verduras en la encimera				
c. Dulces, bollería o galletas				
d. Aperitivos de patatas fritas o snacks				

B. Percepción del entorno alimentario en tiendas

3. ¿Realiza la mayoría de sus compras de alimentación en un solo establecimiento (tienda, supermercado, mercado, cooperativa) o en más de uno? Marque una sola respuesta:

Una tienda/supermercado/mercado/cooperativa	
Dos tienda/supermercado/mercado/cooperativa	
Más de dos	

4. Por favor, indique a continuación el nombre de la o las tiendas/supermercados/mercados/cooperativas donde realiza la <u>mayoría de sus compras:</u>

____

**5. Indique la importancia de los siguientes factores al decidirse por donde compra la mayoría de sus alimentos.**	**Nada**	**Poco importante**	**Algo importante**	**Muy importante**
a. Que esté cerca de mi casa				
b. Que esté cerca o de camino a otros sitios a donde voy				
c. Que mis amigos/familiares compren allí				
d. Variedad de alimentos				
e. Calidad de los alimentos				
f. Precio de los alimentos				
g. Acceso en transporte público				

**6. Donde compra <u>la mayoría de sus alimentos</u>, cómo calificaría los precios de:**	**Baratos**	**Normal**	**Caros**	**Muy caros**	**No sé**
a. Fruta y verdura fresca					

**7. Indique el grado de acuerdo o en desacuerdo con las siguientes afirmaciones. Pensando si en el tipo de tienda donde compra la mayoría de sus alimentos, le resulta fácil comprar:**	**En completo desacuerdo**	**En desacuerdo**	**Ni de acuerdo ni en desacuerdo**	**De acuerdo**	**En completo acuerdo**
a. Fruta y verdura fresca					
b. La oferta es variada					
c. Fruta y verdura enlatada					
d. Productos bajos en grasas					
e. Carnes magras (pollo, pavo,…)					

f. Dulces, bollería o galletas					
g. Aperitivos tipo patatas fritas de bolsa o snacks					
h. Refrescos u otras bebidas azucaradas (bebidas isotónicas, bebidas con zumos concentrados de frutas....)					

8. Piense en el establecimiento <u>donde compra la mayoría de sus alimentos</u> e indique cómo suele ir. Puede seleccionar todas las opciones que correspondan.

a. A pie
b. En bicicleta
c. En autobús u otro medio de transporte público
d. En coche
e. Otra forma (especifique):

En la pregunta 9, se entenderán por:
Alimentos saludables: frutas, verduras, carnes, pescados, huevos, legumbres, aceite de oliva virgen extra,...
Alimentos no saludables: aquellos alimentos que suelen considerarse altos en azúcar, sal, grasas y calorías, tales como los dulces, aperitivos de patata, refrescos, cereales azucarados, bollería industrial, postres, etc.

**9. Indique el grado de acuerdo o en desacuerdo con las siguientes afirmaciones sobre la tienda <u>donde compra la mayoría de sus alimentos:</u>**	**En completo desacuerdo**	**En desacuerdo**	**Ni de acuerdo ni en desacuerdo**	**De acuerdo**	**En completo acuerdo**
a. Veo letreros que me animan a comprar alimentos saludables.					
b. La mayoría de alimentos envasados incluyen la información nutricional en la etiqueta					
c. Los alimentos no saludables suelen estar al inicio o al final de los pasillos					
d. Suelo comprar cosas que están colocadas a la altura de los ojos en la estantería					
e. Hay muchos letreros o expositores que me animan a comprar alimentos no saludables					
f. Suelo comprar alimentos que están cerca de la línea de cajas.					
g. Los alimentaos que están cerca de la línea de cajas suelen ser los alimentos no saludables.					

C. Percepción del entorno alimentario en restaurantes.

**10. Indique con qué frecuencia come o cena fuera o compra comida para llevar en un...**	**Nunca**	**Ocasional mente**	**1 vez al mes**	**Cada 15 días**	**1 vez/ semana**	**2-3 veces semana**	**4-6 veces semana**	**A diario**
a. Restaurante de comida rápida (burguer, pizza, kebab…)								
b. Restaurante de menú o carta								
c. Otro tipo de establecimiento (cafetería, bar, tapas, etc.)								

11. Por favor, indique a continuación el nombre del restaurante/ establecimiento al que acude con más frecuencia: ________________________

12. ¿Cuánto tiempo tardaría en llegar desde su casa hasta el restaurante donde va más a menudo si fuese usted andando?

	10 minutos o menos
	Entre 11 y 20 minutos
	Entre 21 y 30 minutos
	Más de 30 minutos
	Suelo ir en coche, transporte público.

En la pregunta 13, se entenderán por
Opciones saludables: oferta de frutas, verduras, ensaladas, carnes magras, sin rebozar y sin freir, pescados no rebozados y no fritos, huevos sin freir, legumbres, aceite de oliva virgen extra, tamaño pequeño de la ración…
Opciones no saludables: aquellos alimentos que suelen considerarse altos en azúcar, sal, grasas y calorías, tales como los dulces, aperitivos de patata, refrescos, cereales azucarados, bollería industrial, postres, etc.

13. Indique el grado de acuerdo o en descuerdo con las siguientes afirmaciones sobre el establecimiento donde va más a menudo:	En completo desacuerdo	En desacuerdo	Ni de acuerdo ni en desacuerdo	De acuerdo	En completo acuerdo
a. En la carta del restaurante hay muchas opciones saludables.					
b. Me cuesta encontrar una opción saludable cuando voy a comer al restaurante.					
c. Es fácil encontrar opciones saludables que contengan fruta y verdura					
d. El restaurante proporciona información nutricional (como el contenido en calorías o nutrientes) en la carta o en la sala.					
e. Hay letreros y pósters que animan a comer más de la cuenta, a elegir el tamaño de la ración más grande o escoger las opciones no saludables de la carta (combos, tipo "por 1€ más", refresco gratis,…)					
f. La carta o los letreros del restaurante promocionan las opciones saludables o les dan relevancia.					
g. Las opciones saludables son más caras					

D. Opiniones y Hábitos alimentarios

14. A la hora de comprar alimentos, ¿qué importancia tiene para usted…?	Ninguna importancia	Algo de importancia	Mucha importancia
a. El sabor			
b. Que sean saludables			
c. El precio			
d. Comodidad y facilidad de cocinar			
e. Que no engorden			

15. Cuando sale a comer o cenar a un restaurante o compra comida para llevar, ¿qué importancia tiene…?	Ninguna importancia	Algo de importancia	Mucha importancia
a. El sabor			
b. Que sean saludables			
c. El precio			
d. Comodidad y facilidad de acceso			
e. Que no engorden			

16. ¿Con qué frecuencia suele comer los siguientes alimentos?	2 o más veces al día	1 vez al día	5-6 veces a la semana	3-4 veces a la semana	1-3 veces al mes	Menos de una vez al mes
a.Frutas						
b.Verduras (en ensalada o cocinadas)						

17. Cuando va a comprar alimentos, ¿con qué frecuencia se lleva una lista de la compra?

	Nunca
	Ocasionalmente
	A veces
	Normalmente o siempre

E. Preguntas generales sobre usted

18. Indique si es usted... ☐ Hombre ☐ Mujer

19. Indique su edad en años: _____

20. Altura:__metros

21. Peso:_________kilos

22. ¿Cuál es su lugar de nacimiento?

Alicante ☐

España (fuera de Alicante) ☐

Otro país (especifique):_________________Especifique año de llegada:______________

23. Indique su estado civil:

Casado/casada/vivo en pareja ☐ Viudo/viuda ☐

Separado/a o divorciado/a ☐ Soltero/a ☐

24. Cómo describiría su situación laboral actual?

	Trabajador/trabajadora a jornada completa (40 horas a la semana o más, todo el año)
	Trabajador/trabajadora a tiempo parcial
	Desempleado/a, en búsqueda activa de empleo
	Desempleado/a, sin buscar empleo (estudiante, jubilado/a, amo/a de casa, incapacitado/a, etc.)

25. ¿Cuál es su nivel educativo?

- ☐ Sin estudios
- ☐ Educación primaria
- ☐ Educación secundaria
- ☐ Bachillerato
- ☐ Formación Profesional
- ☐ Grado universitario o superior

26. Indique cuál es su estado general de salud.

- ☐ Malo
- ☐ Regular
- ☐ Bueno
- ☐ Muy bueno
- ☐ Excelente

27. ¿Es usted fumador/a?

- ☐ Sí, soy fumador/a
- ☐ Soy exfumador/a
- ☐ No, no he fumado nunca

28. ¿Cómo describiría su nivel de actividad física?

- ☐ Nada activo (vida sedentaria)
- ☐ Moderadamente activo (andar a paso ligero 2-3 veces semana)
- ☐ Entre moderadamente y muy activo (footing o actividades similares 2-3 veces semana)
- ☐ Muy activo (actividad física vigorosa al menos 5 días a la semana)

29. ¿De los siguientes intervalos, entre cuál se encuentran los <u>ingresos netos de su hogar</u> al mes? (sumando todas las fuentes de ingresos de ese hogar)

Ninguno	☐	De 901 a 1.200 €	☐	De 2.701 a 3.600 €	☐
Menor o igual a 600 €	☐	De 1.201 a 1.800 €	☐	Más de 3.600 €	☐
De 601 a 900 €	☐	De 1.801 a 2.700 €	☐	NS/NC	☐

30. ¿Cuántas personas viven en su hogar, además de usted? Nos referimos a cualquier persona que conviva con usted en su casa y comparta la mayor parte de las comidas.

Adultos (18 años o más) ____________________________

Niños (menores de 18 años) _______________________

31. ¿Cómo calificaría su barrio?

- ☐ Urbano (pueblo o ciudad)
- ☐ Suburbano (periferia de pueblo o ciudad; las afueras)
- ☐ Rural o muy rural

32. ¿Podría decirme cuál es su dirección, o en su defecto, el código postal o algún cruce o calle cercana a su casa? ____
